# Bacterial Degraders of Coexisting Dichloromethane, Benzene, and Toluene, Identified by Stable-Isotope Probing

**DOI:** 10.1007/s11270-017-3604-1

**Published:** 2017-10-23

**Authors:** Miho Yoshikawa, Ming Zhang, Futoshi Kurisu, Koki Toyota

**Affiliations:** 1 Geological Survey of Japan, National Institute of Advanced Industrial Science and Technology (AIST), 1-1-1, Higashi, Tsukuba, Ibaraki 305-8567 Japan; 2 Graduate School of Bio-Applications and Systems Engineering, Tokyo University of Agriculture and Technology, 2-24-16, Koganei, Tokyo 184-8588 Japan; 3 Research Center for Water Environment Technology, The University of Tokyo, 7-3-1, Hongo, Bunkyo, Tokyo, 113-8656 Japan

**Keywords:** Aerobic biodegradation, Dichloromethane, Benzene, Multiple volatile organic compounds (multiple VOCs), DNA-stable isotope probing (DNA-SIP)

## Abstract

Most bioremediation studies on volatile organic compounds (VOCs) have focused on a single contaminant or its derived compounds and degraders have been identified under single contaminant conditions. Bioremediation of multiple contaminants remains a challenging issue. To identify a bacterial consortium that degrades multiple VOCs (dichloromethane (DCM), benzene, and toluene), we applied DNA-stable isotope probing. For individual tests, we combined a ^13^C-labeled VOC with other two unlabeled VOCs, and prepared three unlabeled VOCs as a reference. Over 11 days, DNA was periodically extracted from the consortia, and the bacterial community was evaluated by next-generation sequencing of bacterial 16S rRNA gene amplicons. Density gradient fractions of the DNA extracts were amplified by universal bacterial primers for the 16S rRNA gene sequences, and the amplicons were analyzed by terminal restriction fragment length polymorphism (T-RFLP) using restriction enzymes: *Hha*I and *Msp*I. The T-RFLP fragments were identified by 16S rRNA gene cloning and sequencing. Under all test conditions, the consortia were dominated by *Rhodanobacter*, *Bradyrhizobium*/*Afipia*, *Rhizobium*, and *Hyphomicrobium*. DNA derived from *Hyphomicrobium* and *Propioniferax* shifted toward heavier fractions under the condition added with ^13^C-DCM and ^13^C-benzene, respectively, compared with the reference, but no shifts were induced by ^13^C-toluene addition. This implies that *Hyphomicrobium* and *Propioniferax* were the main DCM and benzene degraders, respectively, under the coexisting condition. The known benzene degrader *Pseudomonas* sp. was present but not actively involved in the degradation.

**Electronic supplementary material:**

The online version of this article (10.1007/s11270-017-3604-1) contains supplementary material, which is available to authorized users.

## Introduction

Many volatile organic compounds (VOCs) have been reported as carcinogenic or probably carcinogenic (IARC 2016). VOCs released into the environment can enter the human body through media such as groundwater and soil (ATSDR 2000, 2007), and sites contaminated with VOCs must be remediated. Because VOCs are biodegradable, bioremediation can be a suitable treatment strategy for VOC-contaminated sites (US EPA 2013b), and is especially suitable for large sites (Zhang and Yoshikawa 2016). However, most bioremediation studies have investigated degradation of a single VOC or a VOC and its by-products, rather than multiple VOCs.

Biodegradation of aromatic VOCs has been extensively studied and applied especially on benzene, since many sites are contaminated with benzene (US EPA 2013a, b; Ministry of the Environment, Japan 2016). Aerobic benzene-degrading microorganisms include *Pseudomonas*, *Rhodococcus*, *Nitrosomonas*, *Sphingomonas*, and *Mycobacterium* (Jindrová et al. 2002). In aerobic environments, benzene is degraded into catechol by oxygenase (Gibson et al. 1968). An aliphatic VOC, dichloromethane (DCM), is also relatively easily degraded under aerobic conditions without coexisting VOCs (Capel and Larson 1995). Microorganisms such as *Methyobacterium*, *Hyphomicrobium*, *Bacillus*, *Xanthobacter*, and *Methylopila* degrade DCM by dichloromethane dehalogenase (Muller et al. 2011).

However, real sites such as chemical factories (Priya and Philip 2013), military bases (US EPA 2014), and landfills (US EPA 2015) are often contaminated by multiple VOCs. Several studies have characterized the microbial communities at such sites. For example, at a site contaminated with benzene, toluene, ethylbenzene, and xylenes (BTEX), two possible BTEX degraders, *Acidovorax* and *Propiniovibrio*, were detected by cloning and sequencing analysis (Alfreider and Vogt 2007). Nonetheless, which microorganisms directly degrade specific VOCs in situ remains uncertain.

In this study, we performed a DNA-stable isotope probing (SIP) analysis to identify the degraders of three coexisting VOCs. The three VOCs: DCM, benzene, and toluene, were selected because they are often found in contaminated sites and can be degraded under aerobic conditions (Yoshikawa et al. 2017). Neufeld et al. (2007) remarked that SIP can directly link the phylogenetic and functional information without microbial isolation. However, to the best of our knowledge, degrading microorganisms have not been identified in the presence of multiple coexisting VOCs. To bridge this gap, we combined one ^13^C-labeled VOC (DCM, benzene, or toluene) with the other two unlabeled VOCs, and added the VOCs to a consortium of VOC degraders. DNA extractions from the consortia were followed by gradient fractionation with ultracentrifugation and microbial analysis with terminal restriction fragment length polymorphism (T-RFLP). DNA-SIP can directly reveal the assimilation of each VOC and assists with identifying the degrading bacteria in the multiple VOCs.

## Materials and Methods

### Enrichment Culture

The aerobic degradation of three VOCs (DCM, benzene, and toluene) was performed using an enrichment culture with a soil taken from a contaminated site as a microbial source (Yoshikawa et al. 2016). Initially, 30 mg/L of each VOC was added to the enrichment culture. The aerobic degradations of the three VOCs were maintained by adding DCM, benzene, and toluene and by performing serial transfers to nutrient rich medium at a ratio of 1:5 (*v*/*v*). We named the enrichment culture AE/AE (aerobic/aerobic). After confirming stable degradation, we conducted the following SIP experiments using the AE/AE enrichment culture that no longer contained visible soil particles.

### Incubation with Labeled Benzene, Toluene, and DCM

SIP is a powerful technique for identifying active microorganisms that assimilate particular carbon substrates and/or nutrients into cellular biomass such as DNA. By using a substrate that is enriched with a heavier stable isotope, ^13^C, distinguishing labeled from unlabeled DNAs can be obtained through buoyant density of DNA extract via density gradient centrifugation. As the culture solution for the SIP experiments, we mixed 200 mL of fresh medium that was rich in nutrients (Yoshikawa et al. 2016) and 40 mL of the AE/AE enrichment culture in a 500-mL bottle. The bottle was sealed tightly with a rubber cap and a plastic screw cap, and injected with ^13^C-labeled VOC and/or unlabeled VOCs. We also injected the three unlabeled VOCs into the culture solution as a reference, and into an autoclaved culture solution as a sterile control. The ^13^C-labeled VOCs (purity 99%) were purchased from Cambridge Isotope Laboratories, Inc. (Andover, MA, USA), and the unlabeled VOCs were purchased from Wako Pure Chemical Industries (Osaka, Japan). The ^13^C-benzene and ^13^C-toluene were the ring ^13^C_6_-benzene and ^13^C_6_-toluene, respectively. Each VOC was initially injected at 30 mg/L, and all bottles were prepared in duplicate to confirm reproducibility. The bottles were placed upside down in a dark incubator at 30 °C without shaking. During the incubation period, the VOC concentrations were periodically measured by the headspace analysis method, using a gas chromatograph installed with a flame ionization detector (GC-2014, Shimadzu, Kyoto, Japan) (Yoshikawa et al. 2016).

### DNA Extraction

Culture solutions (2 mL from each bottle) were periodically collected during the VOC degradations. Culture solutions taken from the bottles with added ^13^C-labeled DCM, benzene, and toluene, and the reference bottles, were named AE/AE-13D, AE/AE-13B, AE/AE-13T, and AE/AE-13N, respectively. A portion (0.5 mL) of the collected culture solutions was reserved for DNA extraction using FastDNA® SPIN Kit for Soil (MP Biomedicals, Santa Ana, CA, USA). The kit is applicable to both cultural solutions containing solids and liquid samples (e.g., van der Wielen et al. 2009; Yang et al. 2009). A total of eight DNA extracts from the 0.5 mL portions were collected from the duplicate bottles at the same time. The DNA extracts were mixed, and bacterial communities of the consortia were determined by next-generation sequencing (NGS). The bacteria contributing to the VOC degradations were then elucidated by density gradient fractionation.

### NGS-Based Bacterial DNA Sequencing

The NGS was performed with preparing a library from the DNAs by amplification of targeted region with attachments, and then sequencing adequately amplified library using MiSeq (Illumina Inc., San Diego, CA, USA). The bacterial community was determined by NGS-based amplicon sequencing of the V4 region of 16S rRNA gene. First, PCR was carried out with the universal primers 515F and 806R supplied by FASMAC Co., Ltd. (Atsugi, Kanagawa, Japan). The size of PCR products was analyzed with electrophoresis. Negative control without DNA was simultaneously performed to ensure the quality of experiments. The products of the first PCR were subjected to the second PCR, which attached adaptor sequences to the 5′ end with primers (FASMAC Co., Ltd). These attachments were essential for distinguishing the PCR products under different test conditions. The PCR products were purified with a MinElute® PCR Purification Kit (QIAGEN, Hilden, Germany). The purified PCR products under the four test conditions, distinguished by their different adapter sequences, were equally mixed and analyzed with Miseq (Illumina Inc.).

Sequences that were imperfectly matched to the priming site of the V4 region were eliminated using a Fastx toolkit (ver. 0.0.13.2) (http://hannonlab.cshl.edu/fastx_toolkit/). Sequences with a low-quality score (< 20) and short length (≤ 40) were culled by the sickle (ver. 1.33) tool (https://github.com/najoshi/sickle). After merging the paired end sequences using FLASH (ver. 1.2.10) (https://ccb.jhu.edu/software/FLASH/) (Magoč and Salzberg 2011), the sequences with ≤ 246 and ≥ 260 bp were filtered out with Biopython (http://biopython.org/). Chimeric sequences were removed using usearch (ver. 8.0.1623_i86linux64) (http://drive5.com/usearch/) and Qiime (ver. 1.9.0) (http://qiime.org/) (Caporaso et al. 2010). The non-chimeric sequences with similarities above 97% were grouped into individual operational taxonomic units (OTUs) using mothur (ver. 1.36.1) (http://www.mothur.org/) (Schloss et al. 2009) and MEGA (ver. 7.0.18) (http://www.megasoftware.net/) (Kumar et al. 2016). Phylogenetically affiliated sequences close to the representative sequences were determined by a nucleotide BLAST search (https://blast.ncbi.nlm.nih.gov/Blast.cgi).

### Density Gradient Fractionation by Ultracentrifugation

The DNA extracts obtained under each condition were subjected to density gradient fractionation using ultracentrifugation, as described in Neufeld et al. (2007). DNA (2 ng) in a CsCl/gradient buffer solution was placed in an ultracentrifugation tube and ultra-centrifuged in an Optima L-70K Ultracentrifuge (Beckman Coulter, Brea, CA, USA) at 45,000 rpm for 40 h at 20 °C. Immediately after the spinning had ceased, the DNA fractionated by buoyant density was retrieved from the ultracentrifugation tube by carefully collecting the DNA in the buffer solution at the bottom of the tube. This DNA was divided into 18 fractions. The refractive index of each fraction was measured, and converted to buoyant density with the following equation, *ρ* = a × *η* − b, where *ρ* is buoyant density, *η* is refractive index, and a and b are constants 10.9276 and 13.593 at 20 °C (Birnie 1978), respectively (Noguchi et al. 2014). The CsCl in each fraction was removed as described in Neufeld et al. (2007) with one exception; we added ethachinmate (Nippon Gene, Tokyo, Japan) rather than glycogen to the fraction at a volumetric ratio of 0.01:1.

### DNA Fingerprinting

The DNA samples in each fraction were subjected to T-RFLP. Briefly, the bacterial 16S rRNA genes were PCR-amplified using a primer set of carboxyfluorescein labeled 27F and 907R (Lane 1991). The PCR products with size of approximately 900 bp were confirmed with electrophoresis. The PCR products were purified with MinElute® PCR Purification Kit (QIAGEN), then independently digested with restriction enzymes: *Hha*I (Takara Bio) and *Msp*I (Takara Bio). The digested fragments were separated by capillary electrophoresis (ABI Prism 3100 Genetic Analyzer, Applied Biosystems) as described in Noguchi et al. (2014). The proportions of each T-RF to total T-RFs were quantified via fluorescence intensity.

### Phylogenetic Affiliation of T-RFs

The phylogenetic affiliations of the terminal restriction fragments (T-RFs) were determined by PCR amplification and cloning of the bacterial 16S rRNA gene amplicon from the DNA samples. The DNA samples were selected from the fractions containing T-RFs originated from ^13^C-VOC. The buoyant densities of the fraction for AE/AE-13D and AE/AE-13B were 1.747 and 1.736 g/cm^3^, respectively. For the PCR amplification, we applied the primer set 27F and 1392R (Lane 1991). PCR amplification was confirmed with electrophoresis (band around 1400 bp) and its products were purified with MinElute® PCR Purification Kit (QIAGEN) for cloning. The PCR products were cloned with a TOPO TA Cloning Kit for Sequencing (Thermo Fisher Scientific, Waltham, MA, USA) and *Escherichia coli* HST08 (TAKARA Bio). After blue/white screening to ensure the presence of insert, colonies with correct size of inserts were assayed with colony PCR using the primer set M13F and M13R. The size of insert was confirmed (approximately 1400 bp) with electrophoresis applied to the PCR products. The confirmed PCR products were then purified with MinElute® PCR Purification Kit (QIAGEN), and sequenced (3130xl/3730xl, Thermo Fisher Scientific). Non-chimeric 186 sequences were obtained from the two fractions, and their taxonomic identities were determined by a nucleotide BLAST search. The expected fragment lengths digested with *Hha*I and *Msp*I were determined in the obtained sequences. The phylogenetic affiliations of the observed T-RFs were confirmed by comparing the lengths of the T-RFs and the expected fragments. Previous studies have reported a maximum error of 5 bp in T-RFLP analysis (Clement et al. 1998; Moeseneder et al. 1999; Osborn et al. 2000). Therefore, we divided the fragments differing by less than 5 bp into the same phylogenic classification.

### Quantitative PCR

The DNA samples obtained from each fraction were subjected to quantitative PCR based on their bacterial 16S rRNA genes, using the primer set 338F and 805R, the probe 516F-FAM (Yu et al. 2005), and TaqMan Fast Advanced Master Mix (Thermo Fisher Scientific). Quantitative PCR was carried out with StepOne Plus™ (Thermo Fisher Scientific). Thermal protocol was initially incubated for 2 min at 50 °C and 20 s at 95 °C, followed by 40 cycles of denaturation for 10 s at 95 °C and annealing/extension for 40 s at 60 °C. To ensure the quality and reliability of experiments, DNA samples were quantified in triplicate, and DNA free water was also assayed as a negative control. Calibration curve for the quantitative PCR was made from serial 10-fold dilutions using the 16S rRNA gene from *Escherichia coli* (IAM 12119). The bacterial copy numbers of each phylogenetically affiliated bacterium were calculated from the proportion of each T-RF to total T-RFs and the copy number of the bacterial 16S rRNA gene, and then normalized by the maximum number.

## Results

### Degradation of DCM, Benzene, and Toluene

**Fig. 1:**
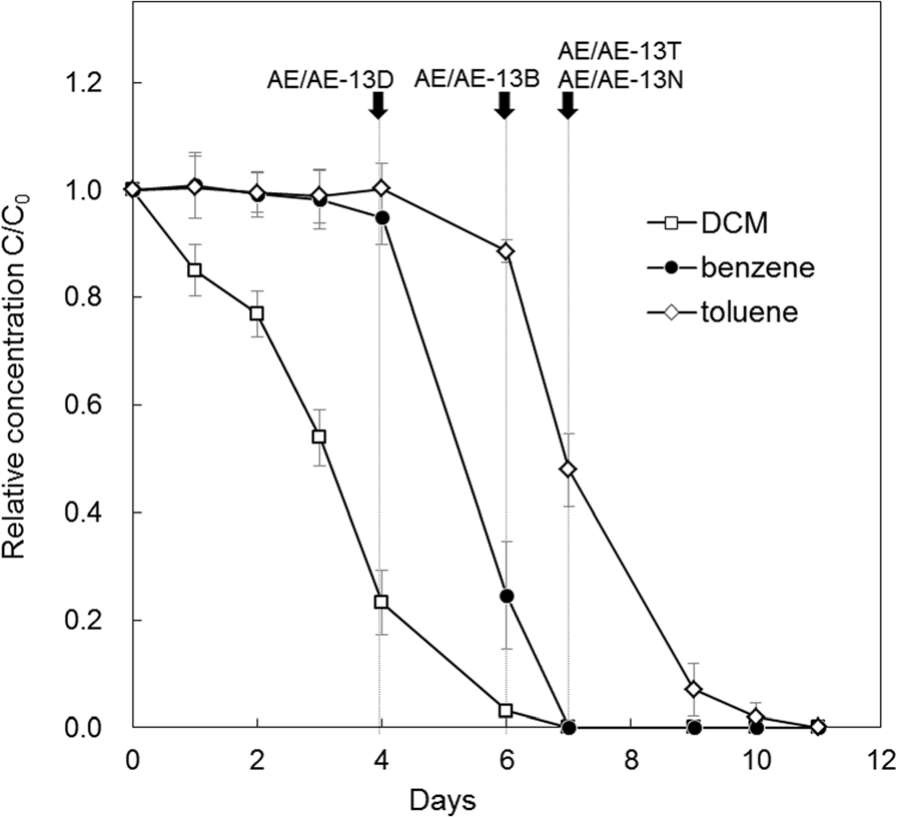
Relative VOC concentrations throughout the test period. Figure 1 plots the average relative concentrations of the labeled and unlabeled VOC and their standard deviations (SDs) in all test conditions. Error bars represent the standard deviation (SD) and arrows indicate the times of sampling the culture solution

DCM, benzene, and toluene were degraded to below their detection limits within 11 days. Figure 1 illustrates the mean and standard deviation of all eight bottles. The degradation started in the order of DCM, benzene, and toluene. The maximum rates of degradation were 11.9 mg-DCM/L/day between days 3 and 4, 11.9 mg-benzene/L/day between days 4 and 6, and 13.4 mg-toluene/L/day between days 6 and 7, respectively. The degradation order was DCM, benzene, and toluene. When each VOC had degraded by around 50–80%, culture solutions were collected for microbial analysis. Specifically, based on their degradation processes, bacterial communities, and degraders for DCM, benzene and toluene in culture solutions were investigated on day 4 (AE/AE-13D), day 6 (AE/AE-13), and day 7 (AE/AE-13T), respectively. As a reference, the culture solution was sampled on day 7 (AE/AE-13N), when degradation of DCM, benzene, and toluene was confirmed. The relative concentrations of DCM, benzene, and toluene in the sterile control to the initial concentrations were 1.01 on day 4, 1.15 on day 6, and 0.86 on day 7, respectively.

### Bacterial Communities in the Culture Solutions

Table 1 lists the OTUs with closest and phylogenetic affiliated sequence, which occupy more than 0.1% in each bacterial community. In all culture solutions under the different test conditions, the representative sequence of the dominant OTU was close to *Rhodanobacter* strain (OTU1) with 100% similarity, and accounted for 43.0–55.2% of the bacterial communities. The OTU1 was followed by three OTUs with high ratios, related to *Bradyrhizobium*/*Afipia*, *Rhizobium*, and *Hyphomicrobium* strains with 100% similarity. These top four OTUs accounted for 79.8–86.2% of the bacterial communities. The culture solution under AE/AE-13D condition contained the highest ratio of *Hyphomicrobium* (11.6%). Sequences grouped into OTU 8 were similar to *Mycobacterium*, and were found under all four test conditions. The sequences close to *Pseudomonas* strains (OTU 12 and 15) were identified from the culture solutions sampled during the early period of incubation (AE/AE-13D and AE/AE-13B), but *Propioniferax* (OTU 14) was identified from the culture solutions sampled during later period of incubation (AE/AE-13B, AE/AE-13T and AE/AE-13N).

**Table 1:** Bacterial communities in the tested culture solutions, characterized by their 16S rRNA gene amplicons

OTU	Organism with closest sequence (accession no.) (similarity, %)	Abundance (%)			
		AE/AE-13D	AE/AE-13B	AE/AE-13T	AE/AE-13N
OTU1	*Rhodanobacter* sp. D1SP217 (KT183544) (100)	43.0	51.4	53.3	55.2
OTU2	*Bradyrhizobium* sp. URHE0020 (LN876515) (100) *Afipia* sp. K43-4AA (KU713085) (100)	14.0	13.2	11.5	10.4
OTU3	*Rhizobium* sp. AT-BI-01 (KX023855) (100)	11.2	13.7	10.6	8.8
OTU4	*Hyphomicrobium* sp. NDB2Meth4 (KY621474) (100)	11.6	7.9	6.7	6.3
OTU5	*Marinobacterium* sp. Sydgv7 (KC462989) (89)	4.1	2.7	3.6	4.2
OTU6	*Mesorhizobium loti* TONO (AP017605) (100)	2.4	2.3	3.3	2.4
OTU7	*Aminobacter aminovorans* LZ1304-3-1 (KX881427) (100) *Mesorhizobium* sp. Lot-his 2 (KX660736) (100)	2.4	1.9	2.7	2.9
OTU8	*Mycobacterium* sp. byf-4 (FJ169473) (100)	2.1	0.7	1.1	0.9
OTU9	*Parvibaculum* sp. TSASRA023 (KJ573556) (96)	1.3	1.3	1.3	2.3
OTU10	*Coxiella* endosymbiont of *Ornithodoros* sp. (KP994802) (93)	1.5	0.9	1.2	1.6
OTU11	*Flavitalea populi* HY-50R (NR_117796) (99)	0.2	0.3	0.7	0.8
OTU12	*Pseudomonas putida* F1 (CP000712) (100)	1.6	0.4	< 0.1	< 0.1
OTU13	*Vermamoeba vermiformis* BCP-EM3VF21-2 (KT185627) (93)	0.1	0.1	0.1	0.8
OTU14	*Propioniferax* sp. P7 (EU109728) (100)	< 0.1	0.2	0.3	0.4
OTU15	*Pseudomonas* sp. Rs81 (AM905941) (100)	0.2	< 0.1	< 0.1	< 0.1
OTU16	*Humibacillus* sp. S3SS536 (KT183563) (99)	0.2	< 0.1	< 0.1	< 0.1
OTU17	Unclassified *Alphaproteobacteria* Ellin6089 (AY234741) (99)	0.1	0.1	< 0.1	< 0.1
OTU18	*Paludisphaera borealis* PT1 (KT372165) (98)	0.1	< 0.1	< 0.1	< 0.1
OTU19	*Thiobacillus thioparus* NZ (KC542801) (100)	0.1	< 0.1	< 0.1	< 0.1

AE/AE-13D: sampling time, day 4; degrading ratios: DCM 75%, benzene 3%, and toluene 0%. AE/AE-13B: sampling time, day 6; degrading ratios: DCM 97%, benzene 75%, and toluene 11%. AE/AE-13T: sampling time, day 7; degrading ratios: DCM 100%, benzene 100%, and toluene 51%. AE/AE-13N: sampling time, day 7; degrading ratios: DCM 100%, benzene 100%, and toluene 53%

### Comparison of DNA Buoyant Density Distributions in AE/AE-13D and AE/AE-13N

**Fig. 2:**
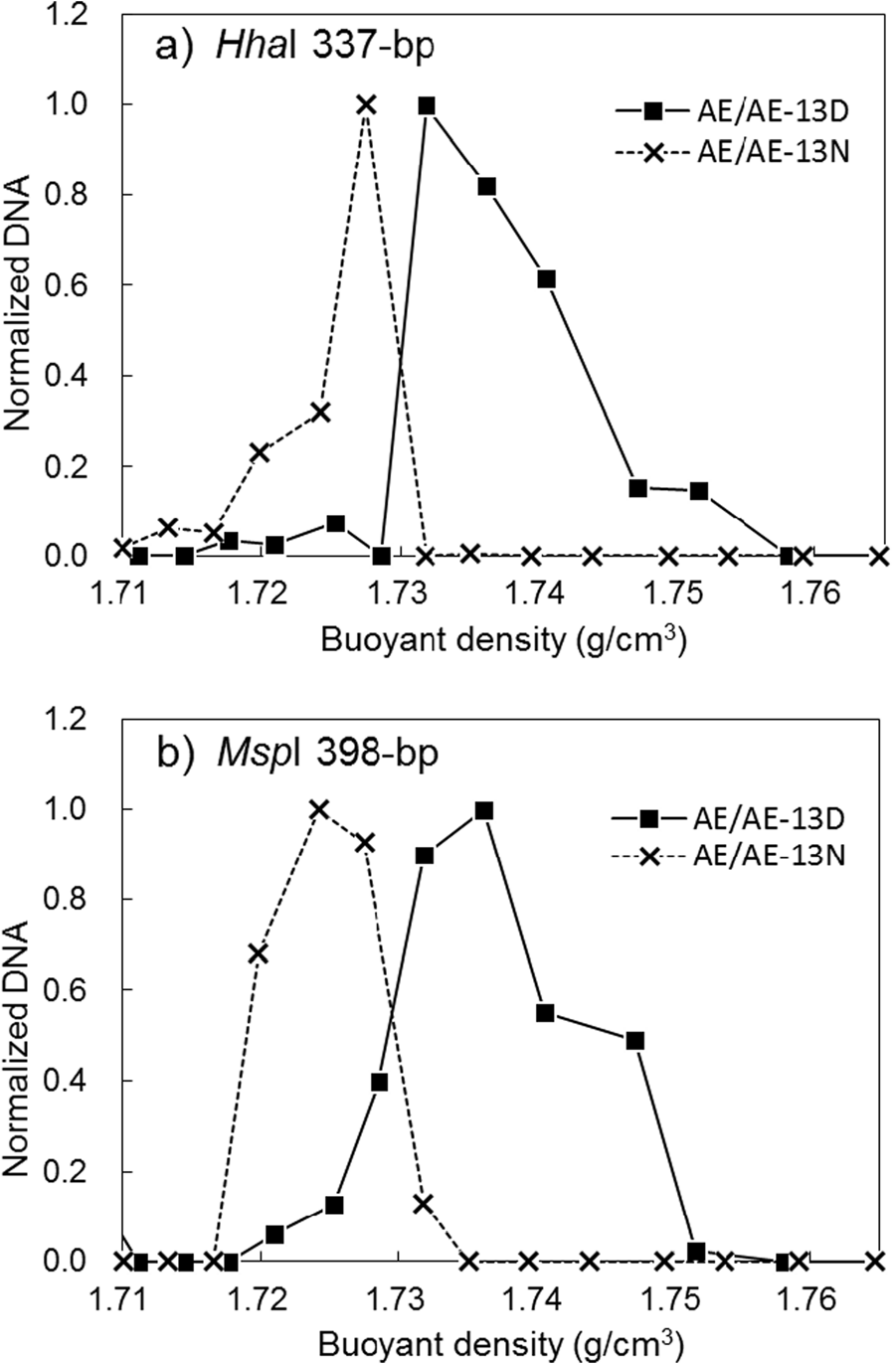
Normalized DNA distributions of T-RFs in fractions from the ^13^C-DCM labeled test (AE/AE-13D) and the unlabeled test (AE/AE-13N). **a** 337-bp T-RF digested with *Hha*I. **b** 398-bp T-RF digested with *Msp*I

In the ^13^C-DCM labeled AE/AE-13D, the normalized DNA distribution of a 337-bp T-RF digested with *Hha*I shifted toward heavier fractions from the distribution in unlabeled AE/AE-13N (Fig. 2a). The normalized DNA distribution of a 398-bpT-RF digested with *Msp*I also shifted toward heavier fractions in AE/AE-13D (Fig. 2b). The T-RFs in both heavier fractions (≥ 1.732 g/cm^3^) were more abundant in AE/AE-13D than in AE/AE-13N.

### Comparison of DNA Buoyant Density Distribution Between AE/AE-13B and AE/AE-13N

**Fig. 3:**
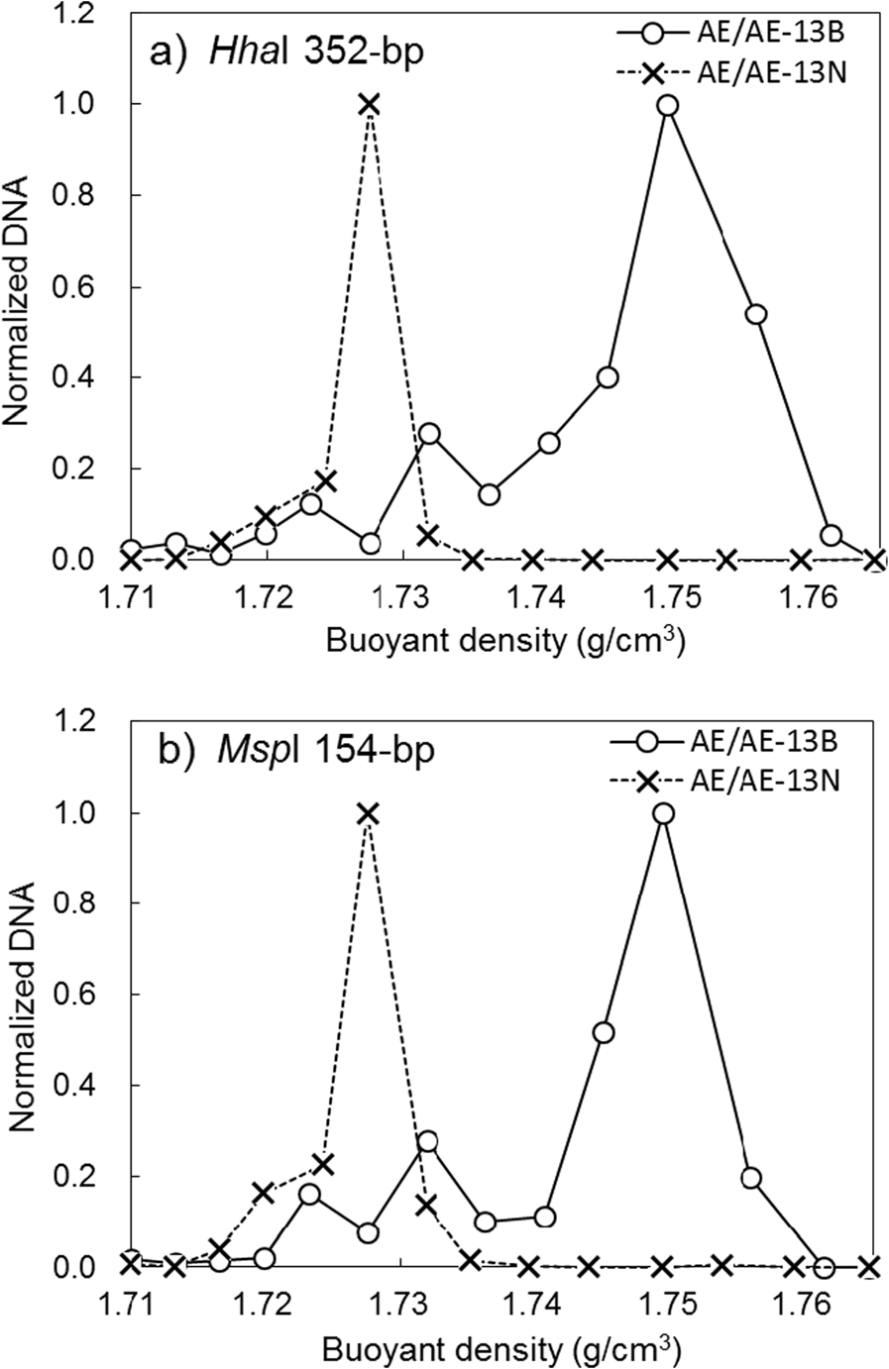
Normalized DNA distributions of T-RFs in fractions from the ^13^C-benzene labeled test (AE/AE-13B) and unlabeled test (AE/AE-13N). **a** 352-bp T-RF digested with *Hha*I. **b** 154-bp T-RF digested with *Msp*I

The normalized DNA distributions of five T-RFs shifted toward heavier fractions in the ^13^C-benzene labeled AE/AE-13B from those in AE/AE-13N; 52-bp, 352-bp, and 784-bp fragments digested with *Hha*I, and 120-bp and 154-bp fragments digested with *Msp*I. The 352-bp T-RF shifted obviously to heavier fractions (Fig. 3a). A remarkable shift was also confirmed in the 154-bp T-RF digested with *Msp*I (Fig. 3b). The distribution peaks of the 352-bp and 154-bp T-RFs in AE/AE-13N and AE/AE-13B appeared at buoyant densities of 1.727 and 1.749 g/cm^3^, respectively. The distribution shifts of other T-RFs were smaller, ranging from 1.727 g/cm^3^ in AE/AE-13N to 1.732 g/cm^3^ in AE/AE-13B, as shown in supplemental material (Table S1). In the following discussion, we focus on the 352-bp T-RF digested with *Hha*I and the 154-bp digested with *Msp*I, which exhibited dramatic shifts to heavier fractions in AE/AE-13B.

### Comparison of DNA Buoyant Density Distribution Between AE/AE-13T and AE/AE-13N

In the ^13^C-toluene labeled AE/AE-13T, none of the DNAs originating from the T-RFs digested by *Hha*I and *Msp*I shifted obviously from their distributions in AE/AE-13N (Table S1).

### Phylogenic Affiliations of T-RFs

To estimate the phylogenic affiliations of the shifted fragments, we cloned and sequenced the 16S rRNA gene sequences in the heavier DNA fractions. The terminal restriction fragment lengths of the clones were estimated in silico and compared with those of the T-RFs (Table 2). The obtained clonal sequence AE/AE-13D-05-001 was highly similar to *Hyphomicrobium* sp. NDB2Meth4, so cutting with *Hha*I and *Msp*I, should yield 341 and 403 bp T-RFs, respectively. The sequence completely matched a *Hyphomicrobium* sequence affiliated in our previous study (Yoshikawa et al. 2016). Therefore, the T-RFs obtained in AE/AE-13D (337 and 398 bp after digestion with *Hha*I and *Msp*I, respectively) were identified as *Hyphomicrobium.* Another clone in the sample, with close similarity to *Caulobacter mirabilis* 0112ALTE9 (98%), should also produce a 402-bp T-RF when cut with *Msp*I, so the fragments in the observed 398-bp T-RF might be derived from both *Caulobacter mirabilis* and *Hyphomicrobium*. The clonal sequences with high similarity to *Propioniferax* sp. P7 (namely, AE/AE-13B-07-011), should be digested to 355 and 158 bp with *Hha*I and *Msp*I, respectively. The clonal sequence matched a *Propioniferax* sequence determined in our previous study (Yoshikawa et al. 2016), with 100% similarity. Based on these results, we affiliated the fragments that were mass-shifted in AE/AE-13B (the 352-bp and 154-bp T-RFs digested with *Hha*I and *Msp*I, respectively) with *Propioniferax*.

**Table 2:** Comparison of fragment lengths determined in T-RFLP and predicted by sequencing

T-RFLP analysis		Cloning analysis		
Culture solution	Restriction enzyme/T-RF length (bp)	Clone ID	Restriction enzyme/expected T-RFs (bp)	Organism with closest sequence (accession no.) (similarity, %)
AE/AE-13D	*Hha*I/337	AE/AE-13D-05-001	*Hha*I/341	*Hyphomicrobium* sp. NDB2Meth4 (KY621474) (99)
	*Msp*I/398		*Msp*I/403	
AE/AE-13B	*Hha*I/352	AE/AE-13B-07-011	*Hha*I/355	*Propioniferax* sp. P7 (EU109728) (99)
	*Msp*I/154		*Msp*I/158	

## Discussion

### Bacterial Degraders of DCM

*Hyphomicrobium* sp. was found to assimilate DCM by the SIP analysis, indicating that *Hyphomicrobium* a known DCM degrader also works under the co-mingled condition with benzene and toluene. Applying the SIP method, Kasai et al. (2006) and Noguchi et al. (2014) identified microorganisms associated with the anaerobic degradation of benzene, and we adopted a similar approach to initially identify DCM degraders under aerobic test conditions without isolation. Strains of *Hyphomicrobium* has been isolated from contaminated soils (Stucki et al. 1981), groundwater (Nikolausz et al. 2005), and sludge (Ottengraf et al. 1986; Nikolausz et al. 2005). *Hyphomicrobium* are known to aerobically degrade DCM in the absence of other VOCs (Muller et al. 2011), and *Hyphomicrobium* sp. KDM2 can degrade DCM under anaerobic condition (Nikolausz et al. 2005). However, the sequence of *Hyphomicrobium* (AE/AE-13D-05-001) in this study was distinct from previously known *Hyphomicrobium* that degraded DCM (up to 96% similarity). The sequence of AE/AE-13D-05-001 was close to *Hyphomicrobium* sp. LAT3 that can degrade methyl chloride (Borodina et al. 2005) with 99% similarity. *Hyphomicrobium* which degrades benzene, toluene, ethylbenzene, and xylene has not been reported so far.

The DCM degradability in multiple-VOC environments has remained unclarified. The present results demonstrated that *Hyphomicrobium* started and completed degradation of DCM in the presence of benzene and toluene, two chemicals that cannot be assimilated by *Hyphomicrobium*. The sequence of *Hyphomicrobium* (AE/AE-13D-05-001) was closest to that of *Hyphomicrobium* sp. NDB2Meth4 (KY621474) with 99% similarity. The strain NDB2Meth4 was isolated from a coal seam gas extraction bore well. In general, the subsurface environment contains a variety of aliphatic and aromatic compounds (Orem et al. 2014). In addition, *Hyphomicrobium* sp. NDB2Meth4 has toluene tolerance protein (WP_072377846). The *Hyphomicrobium* affiliated in the present study might be tolerant to coexisting benzene and toluene, and kept the degradability for DCM.

### Bacterial Degraders of Benzene

Compared with the shifts in Fig. 2 for DCM, the shifts in Fig. 3 for benzene are bigger. Although the concentrations of both contaminants were set at the same level (30 mg/L), the labeled carbon numbers of benzene are six and just one in DCM. This might be a reason that results in bigger shifts for benzene. The peak shift in AE/AE-13B indicated *Propioniferax* sp. is a key microorganism that contributed to degradation of benzene. Although this organism has not yet been isolated as a benzene degrader, it can be a degrader or a principal microorganism that is responsible for carbon assimilation during benzene degradation. A strain originally isolated from human skin (Pitcher and Collins 1991) was proposed as a novel genus *Propioniferax* by Yokota et al. (1994). *Propioniferax* produces propionic acid from glucose (Pitcher and Collins 1991; Yokota et al. 1994). *Propioniferax* is facultatively anaerobic bacteria, though substantial growth of the bacteria occurs aerobically. The cloned colony sequence from AE/AE-13B-07-011 and the representative sequence from OTU14 in NGS shared 98% identity with *Propioniferax* sp. RO1. Strain RO1, which originated from paddy field soil and was isolated under anaerobic condition, produces electricity from lactate (Rubaba et al. 2013). Although our previous study (Yoshikawa et al. 2016) suggested the possibility, this study directly confirmed that *Propioniferax* involved in degrading benzene based on its assimilation by using DNA-SIP technique.

*Pseudomonas* existed in the consortia, as evidenced by NGS, but did not directly degrade benzene according to the DNA-SIP results. Since *Pseudomonas* is one of well-known degraders of benzene, this result means that under the co-mingled condition, the degrader is not the same as that being understood under single contaminant condition. The representative sequence from OTU12 in Table 1 shows high sequence similarity with *P*. *putida* F1 (100%). In addition, a representative sequence from OTU15 perfectly matched the sequence of *Pseudomonas* sp. Rs81. Strains F1 and Rs81 are benzene-degrading bacteria (Gibson et al. 1968; Fahy et al. 2008). The results suggest that competitive interactions might occur between *Pseudomonas* spp. and other microorganisms during the degradation of benzene. *Pseudomonas* spp. might be outcompeted by the competing microorganisms due to nutrient competition, space competition, production of antimicrobial compounds, and disruption of the signals (Hibbing et al. 2010). The findings illustrate that to understand the compound-degrading microorganisms in microbial communities, we must employ techniques (such as DNA-SIP) that directly analyze the substrate assimilation in situ.

### Bacterial Degraders of Toluene

The uptake of ^13^C-toluene to form bacterial DNA was not supported by the SIP analysis, as no shift from normalized DNA appeared in AE/AE-13T. This lack of uptake could be caused by assimilation time lag. Toluene is assimilated through steps in the toluene degradation pathway (van Agteren et al. 1998). The *Mycobacterium* detected by NGS was grouped into OTU8. The representative sequence of OTU8 is 100% similar to the known toluene-degrading *Mycobacterium* strains byf-4 (FJ169473) (Zhang et al. 2013) and T103 (Tay et al. 1998). Zhang et al. (2013) found that after a 70% toluene reduction by *Mycobacterium* sp. byf-4, the toluene by-product 3-methylcatechol remained and accounted for 20% of the decrement. After four subsequent enzymatic steps, the by-product 3-methylcatechol is converted to acetaldehyde and pyruvate (van Agteren et al. 1998), which enter the tricarboxylic acid cycle through acetyl CoA. Although *Mycobacterium* might degrade toluene, they did not assimilate toluene over the sampling period in the present study.

## Conclusions

The DNA-SIP analysis demonstrated that *Hyphomicrobium* assimilates DCM in culture solutions containing multiple VOCs (DCM, benzene, and toluene). Under this co-mingled condition, *Propioniferax* was identified as a key bacterium that contributes to benzene degradation. The results suggested that the microorganisms maintain degradability of the VOCs. The well-known benzene degrader *Pseudomonas* existed in the consortium but did not degrade benzene in the mixed VOC.

## Electronic Supplementary Material

ESM 1 (DOCX 28 kb)
