# Supplementary material for: Bacterial Degraders of Coexisting Dichloromethane, Benzene, and Toluene, Identified by Stable-Isotope Probing
Source: Water Air Soil Pollut. 2017 Oct 23;228(11):418. doi: 10.1007/s11270-017-3604-1 (PMC5653698; doi:10.1007/s11270-017-3604-1)
Supplement: Supplementary file 1 — (DOCX 28 kb) [file 11270_2017_3604_MOESM1_ESM.docx]

**Supplemental Material**

**Bacterial degraders of coexisting dichloromethane, benzene and toluene, identified by stable-isotope probing**

Miho Yoshikawa^1, 3^, Ming Zhang^1^, Futoshi Kurisu^2^, and Koki Toyota^3^

^1^ Geological Survey of Japan, National Institute of Advanced Industrial Science and Technology (AIST), 1-1-1, Higashi, Tsukuba, Ibaraki 305-8567, Japan

^2^ Research Center for Water Environment Technology, The University of Tokyo, 7-3-1, Hongo, Bunkyo, Tokyo 113-8656, Japan

^3^ Graduate School of Bio-Applications and Systems Engineering, Tokyo University of Agriculture and Technology, 2-24-16, Koganei, Tokyo 184-8588, Japan

E-mail: m.zhang@aist.go.jp; Tel: +81-29-861-3943; Fax: +81-29-861-8773

**Supplemental Material for Results**

**Table S1** Buoyant density of fraction with highest normalized DNA originated from a T-RF

| Restriction enzyme | T-RF (bp) | Buoyant density of fraction with highest normalized DNA (g/cm^3^) | | | |
| --- | --- | --- | --- | --- | --- |
|  |  | AE/AE-13D | AE/AE-13B | AE/AE-13T | AE/AE-13N |
| *Hha*I | 52 | 1.729 | 1.732 | 1.727 | 1.727 |
|  | 337 | 1.732 | 1.723 | 1.724 | 1.727 |
|  | 352 | 1.729 | 1.749 | 1.727 | 1.727 |
|  | 784 | 1.725 | 1.732 | 1.727 | 1.727 |
| *Msp*I | 120 | 1.729 | 1.732 | 1.727 | 1.724 |
|  | 154 | 1.729 | 1.749 | 1.727 | 1.727 |
|  | 398 | 1.736 | 1.723 | 1.727 | 1.724 |

+0.004 0.008 0.012 0.016 0.020 　　 0.024 g/cm^3^

Colored cells show the buoyant densities that are greater than those of the reference AE/AE-13N (rightmost column).
